# Annexin A protein family: Focusing on the occurrence, progression and treatment of cancer

**DOI:** 10.3389/fcell.2023.1141331

**Published:** 2023-03-03

**Authors:** Huhu Zhang, Zhe Zhang, Tingting Guo, Guang Chen, Guoxiang Liu, Qinghang Song, Guichun Li, Fenghua Xu, Xiaolei Dong, Fanghao Yang, Can Cao, Di Zhong, Shuang Li, Ya Li, Mengjun Wang, Bing Li, Lina Yang

**Affiliations:** ^1^ Department of Genetics and Cell Biology, Basic Medical College, Qingdao University, Qingdao, China; ^2^ Department of Traditional Chinese Medicine, The People’s Hospital of Zhaoyuan City, Yantai, China; ^3^ Health Science Center, Qingdao University, Qingdao, China; ^4^ Department of Hematology, The Affiliated Hospital of Qingdao University, Qingdao, China

**Keywords:** annexin a protein family, structure, cancer progression, biomarker, treatment, inhibitor

## Abstract

The annexin A (ANXA) protein family is a well-known tissue-specific multigene family that encodes Ca^2+^ phospholipid-binding proteins. A considerable amount of literature is available on the abnormal expression of ANXA proteins in various malignant diseases, including cancer, atherosclerosis and diabetes. As critical regulatory molecules in cancer, ANXA proteins play an essential role in cancer progression, proliferation, invasion and metastasis. Recent studies about their structure, biological properties and functions in different types of cancers are briefly summarised in this review. We further discuss the use of ANXA as new class of targets in the clinical diagnosis and treatment of cancer.

## 1 Introduction

ANXs are proteins that are encoded by a highly conserved multigene family and are widely found in animals, plants and protists (Geisow et al., 1987). In 1978, ANXs were given different names based on their different biochemical properties and were divided into five categories: A, B, C, D and E^2^. The ANXA protein family in humans contains 12 species, ANXA1– ANXA11 and ANXA13 (Croissant et al., 2021), all of which are small soluble proteins. They are uniquely expressed in specific tissues; for example, ANXA3 induces human umbilical vein endothelial cell migration and tubulogenesis (Park et al., 2005), ANXA4 is specifically expressed in cochlear and vestibular hair cells (Li et al., 2021a), while, while ANXA5 participates in the activation of immune T cells (Hu et al., 2020).

Tissue-specific ANXA proteins exist in different cancers, including hepatocellular carcinoma (Dong et al., 2014), gastric cancer (Wang et al., 2013), breast cancer (Sharma et al., 2012), ovarian cancer (Manai et al., 2020) and lung cancer (Zhou et al., 2021a). In addition, numerous studies have indicated that ANXA is expressed irregularly in disparate cancers. For instance, ANXA2 is elevated in breast cancer and stimulates fibrinolytic enzyme production, which results in angiogenesis and metastasis. In contrast, ANXA6 expression is downregulated in primary gastric cancer tissues and directly inactivates the Ras/MAPK signalling pathway (Wang et al., 2013) to inhibit gastric cancer growth. Aside from ANXA2 and ANXA6, other ANXA family proteins can be used as markers of tumour occurrence and development as they affect a variety of signal pathways, such as cancer cell invasion, migration, apoptosis and autophagy. Furthermore, some ANXA proteins cause drug resistance and are thus potential therapeutic targets for tumours. A discussion of ANXA and their inhibitors may provide better strategies for the early diagnosis, clinical treatment and prognosis of cancer.

## 2 The molecular structure of ANXA protein family

**FIGURE 1:**
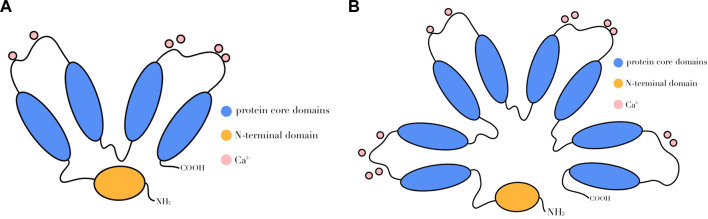
Schematic diagram of ANXA protein structure. ANXA consists of two regions, one of which is the C-terminus, the characteristic highly conserved core structural domain that usually consists of four repetitive sequences **(A)**, while ANXA6 has eight repetitive sequences **(B)**, each of which contains a calcium-binding motif. Another region is the N-terminal, where the amino acid arrangement order and length of each ANXA varies.

The ANXA protein family contains structural and biological features. Structurally, the C-terminal region is conserved, with most membrane-bound proteins consisting of four fragments of around 70 amino acids each, which are internally and intrinsically homologous to the membrane-bound proteins. An exception is the ANXA6 protein, which has eight core fragments that form a high alpha helix and a closely spaced disc-like structure (Figure 1) (Li et al., 2022).

The biological functions of the superfamily members differ depending on the N-terminal domain, which is where most of their post-translational modifications occur (Wang et al., 2019). In biology, the ANXA protein family is a group of calcium-dependent phospholipid-binding proteins, so called because they only bind to phospholipid membranes with conformational changes in the presence of Ca^2+12^. This binding is, however, reversible. The removal of Ca^2+^ reveals the N-terminus hidden in the centre of the C-terminus (Rintala-Dempsey et al., 2008). The coordination of ANXA to calcium ions requires the involvement of water molecules to ensure the two ligands remain stable (Rintala-Dempsey et al., 2008). Besides Ca^2+^, the N-terminal of ANXA has multiple binding sites, including interacting partners. For instance, the S100 family, which comprises electron chiral Ca^2+^ binding proteins (Bharadwaj et al., 2013), functions to regulate cell proliferation, membrane transport and calcium homeostasis (Donato et al., 2013). The S100–ANXA interaction has been shown to play a role in membrane fusion by linking an S100 protein to two ANXA proteins on a phospholipid membrane (Rintala-Dempsey et al., 2008). Because of these different sequences in the N-terminal, ANXA members display distinctive properties and diverse functions. ANXA3 has a strong binding ability to phospholipids *via* the substitution of classical Trp-5 with alanine (Hofmann et al., 2000), while ANXA1 phosphorylates diverse protein residues *via* its N-terminal kinases characteristic (Rosengarth et al., 2001). Generally, these subtle changes do not affect their basic structure of ANXA proteins.

## 3 The role of ANXA protein family in cancer progression

### 3.1 The role of ANXA protein family in cell proliferation

The ANXA protein family can inhibit tumour proliferation by affecting cell membrane and cytoskeleton formation. In nasopharyngeal carcinoma, decreased ANXA1 leads to the upregulation of S100A9/vimentin, which increases tumour invasion by regulating the function of the cytoskeletal proteins (Xiao et al., 2017). When ANXA2 expression is effectively inhibited, the growth and motility of both the human colorectal cancer cell line CACO2 and human hepatocellular carcinoma cell line SMMC7721 are significantly reduced, and the motility-associated microstructures, such as pseudopods and filamentous pseudopods, as well as the polymerisation of microfilaments and microtubules, are significantly inhibited (He et al., 2019).

**FIGURE 2:**
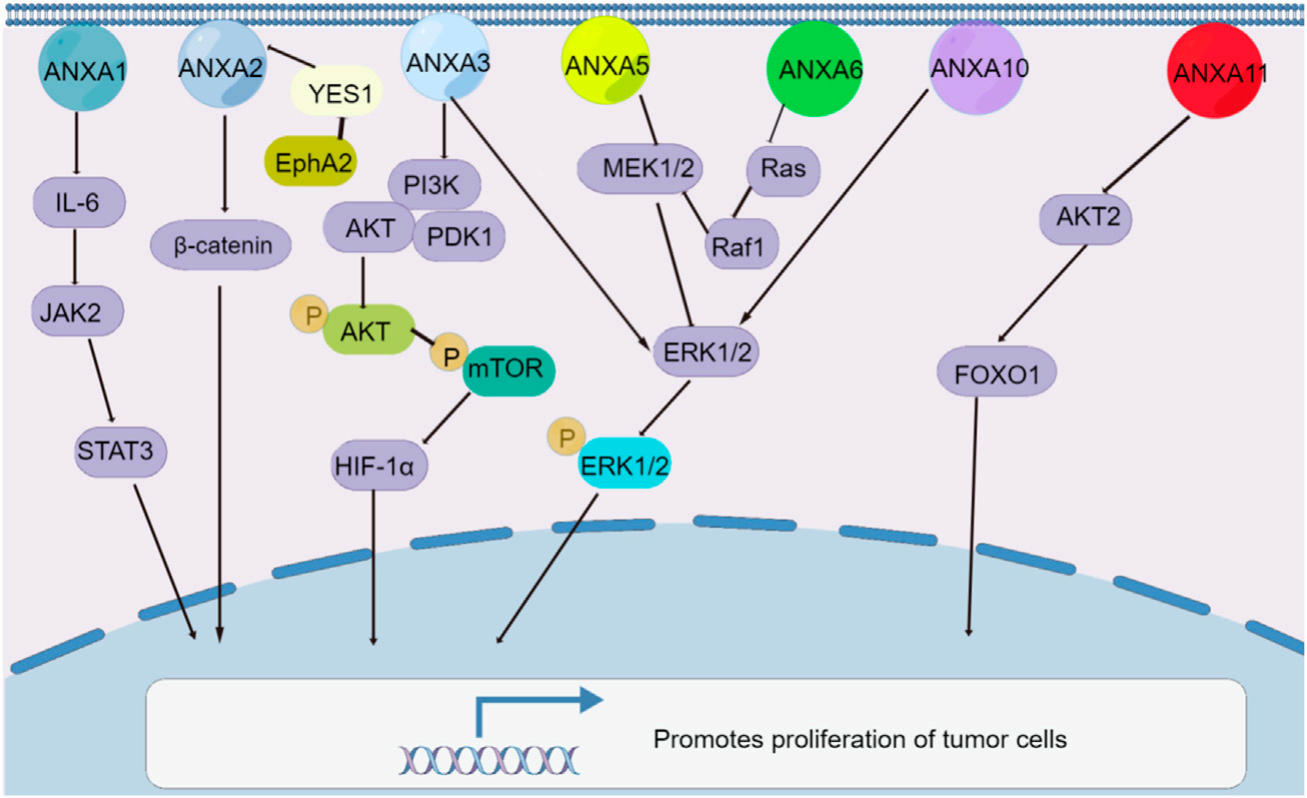
Signaling pathways of ANXA protein family regulating tumor cell proliferation. Except for ANXA6, the rest of the ANXA protein family was overexpressed and promoted the proliferation of tumor cells.

The members of the ANXA protein family play the opposing roles of promoting and inhibiting tumour proliferation through their participation in or activation of signalling pathways. The exceptions are ANXA5, ANXA6, ANXA10 and ANXA11, which play roles in several tumour types, while other ANXA members promote tumour cell proliferation (Grewal et al., 2010; Li et al., 2018a; Liu et al., 2019; Liu et al., 2020; Liu et al., 2021a; Zhou et al., 2021a; Zhou et al., 2021b; Gu et al., 2021; Huang et al., 2021; Ju et al., 2021; Wei and Zhu, 2021). In hepatocellular carcinoma, the overexpression of ANXA2 (Zhang et al., 2013), ANXA3 (Guo et al., 2021a), ANXA4 (Liu et al., 2017), ANXA5 (Sun et al., 2018) and ANXA11 (Liu et al., 2019) promotes proliferation because ANXA2–ANXA5 affect Wnt/β-catenin, MEK-ERK, PI3K/AKT-HIF-α and other pathways. It is worth mentioning that ANXA4 may be a key factor in hepatocellular carcinoma tumorigenesis (Liu et al., 2017), as downregulating ANXA4 expression can inhibit hepatocellular carcinoma proliferation and tumorigenesis *in vitro* and *in vivo*. Similarly, in lung cancer, the overexpression of ANXA8 activates the EGFR/AKT/mTOR signalling pathway to promote proliferation (Zhou et al., 2021a), while in thyroid cancer, increased ANXA1 promotes thyroid cancer proliferation by mediating IL-6/JAK2/STAT3 (Zhao et al., 2021). In contrast, the overexpression of the ANXA10 gene significantly inhibits HepG2 cell proliferation *in vitro*, and its downregulation inhibits papillary thyroid cancer proliferation; it acts directly on TSG101 by inactivating the MAPK/ERK signalling pathway (Wei and Zhu, 2021). In gastric cancer, different ANXA proteins show either proliferation promoting or inhibiting effects. For instance, ANXA2 directs the interaction of the protein YES1 and involves the EphA2-YES1-ANXA2 pathway (Mao et al., 2021), which promotes gastric cancer progression and metastasis, while ANXA6 (Wang et al., 2013) suppresses gastric cancer progression by inhibiting Ras/MAPK signalling. Moreover, increased ANXA5 inhibits cervical cancer proliferation by regulating the expression of Bcl-2 and Bax (Li et al., 2018a). A large number of studies have demonstrated that ANXA proteins are involved in the regulation of tumour proliferation, and these studies may provide new ideas and directions for research on tumour prevention and targeted therapies depending on the understanding of their role in the pathogenesis of malignant tumours (Figure 2).

### 3.2 The role of the ANXA protein family in the cell cycle

**FIGURE 3:**
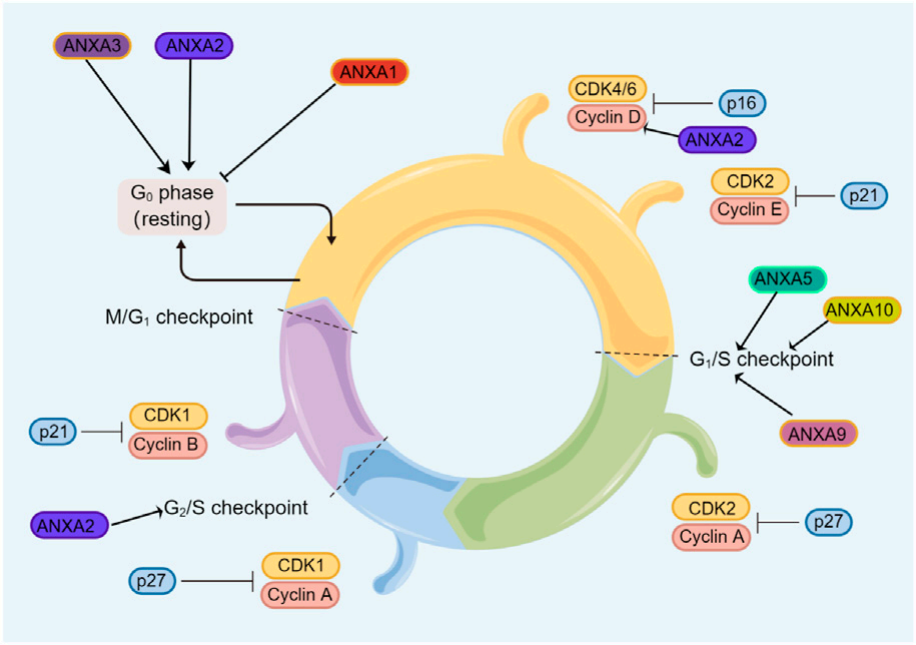
Regulation of the ANXA protein family in the cell cycle. Members of the ANXA protein family promotes tumor cell cycle progression.

The cell cycle is a complex process involving many regulatory proteins that guide cells through specific sequences of events that culminate in the production of two daughter cells (Schafer, 1998). The ANXA protein family plays critical roles in cell cycle regulation in a complex manner (Figure 3). ANXA1 promotes G_0_/G_1_ phase cyclic arrest in leukaemic K562 and U937 cells and promotes apoptosis in leukaemia (Tsai et al., 2013; Sabran et al., 2019). In one study, silencing ANXA1 in hypopharyngeal carcinoma not only significantly decreased the proportion of G_0_/G_1_ phase cells, but significantly increased the proportion of S phase cells, which enhanced cell activity and promoted cell cycle progression (Li et al., 2021b).

ANXA2 regulates c-Myc expression by binding to the 5′UTR of its mRNA in a Ca^2+^-dependent manner at the two pseudoknots of the internal ribosome entry site. Thus, downstream cyclinD1, a cell cycle regulator protein, is affected, which leads to cell proliferation and a high proportion of S-phase cells (Strand et al., 2021; Sun and Zhang, 2021). The downregulation of ANXA2 stalls cells in the G_1_ phase or S-G_2_/M phase in gastric cancer (Sun et al., 2013) and glioblastoma multiforme (Maule et al., 2016), respectively. A study showed that the ANXA3 silencing group had a lower proliferation rate in breast cancer and a higher cell ratio in the G_0_/G_1_ compared to the control group (Li et al., 2018b). In oncogenic human papillomavirus-positive non-small cell lung cancer, ANXA4 is mediated by HPV16E7 and HPV16E6/E7, promotes cell proliferation and regulates the cell cycle and mitosis (Ciotti et al., 2009). Meanwhile, the combination of epigallocatechin gallate and quercetin enhance ANXA5 to effectively inhibit colorectal cancer arrest at the G_1_ stage (Xue et al., 2009; Al-Ghamdi et al., 2021). Furthermore, the overexpression of ANXA9 has been found to increase the migration of gastric cancer and form larger and more cell clones, which decrease in the G_1_ phase and increase in the S and G_2_ phases (Zhou et al., 2021b). ANXA10 is frequently found to be highly expressed in human oral cancer, and this high expression may promote G_1_ phase cell cycle progression by activating the ERK/MAPK signalling pathway, which in turn results in the reduced expression of member-dependent kinases of cell cycle proteins (Shimizu et al., 2012). To date, most studies have described ANXA protein family members as promoting the cell cycle in cancer, although little is known about the associated mechanisms. Future investigations regarding the impact of ANXA proteins on the regulation of the cell cycle would certainly inspire more exploration of their biological effects.

### 3.3 The role of ANXA protein family in invasion and metastasis

Invasion describes the occupation of adjacent normal tissues by cancer cells after leaving the tumour parent body, and metastasis is the spread of tumour cells from the primary site to distant organs (Hart and Fidler, 1980). Increasing evidence has elucidated the critical role of the ANXA protein family in cancer invasion and metastasis (Xu et al., 2015). Proteomics technology has revealed that ANXA1 mainly regulates the processes related to tumour cell skeletal remodelling and immune responses, thus affecting the invasive migration of cancer cells (Tu et al., 2017). Of particular interest, ANXA1 plays dual roles with regard to invasion and metastasis among different tumour cells (Liu et al., 2014; Swa et al., 2015). A study showed that the inhibition of ANXA1 in nasopharyngeal carcinoma increased the trauma-healing ability of the cells, and the activation of the ANXA1 gene decreased the trauma-healing rate by 70%. Conversely, in the mouse mammary tumour cell line 168FARN, the knockdown of ANXA1 inhibited the invasion and metastasis of breast cancer (Tu et al., 2017), with less expression of epithelial–mesenchymal transition (EMT) markers, wave proteins and myosin light-chain kinase.

In addition to ANXA1, the ANXA proteins ANXA2, ANXA9 and ANXA10 are associated with tumour cell adhesion, invasion and metastasis (Xu et al., 2015; Yu et al., 2018; Sun et al., 2019). Once ANXA2 binds to fibrinogen and tissue-type fibrinogen activators, it can activate metalloproteinase (MMPs), which will in turn disrupt the integrity of the extracellular matrix, resulting in tumour invasion and metastasis (Xu et al., 2015). Accordingly, in gastric cancer HGC-27 cells, the inhibition of ANXA2 was found to significantly reduce the migration and secretion of MMPs and inhibit cancer invasion and metastasis (Han et al., 2017). Similarly, Shi et al. (2020) Found that TAGLN2–ANXA2 interaction induces invasion and metastasis in hepatocellular carcinoma. In oesophageal cancer, the overexpression of ANXA2 promotes ESCC cell invasion *in vitro* and metastasis *in vivo* through the activation of the MYC-HIF-1α-VEGF cascade (Ma et al., 2018). More meaningfully, some clinical studies have shown the relationship between the expression levels of ANXA2 and the invasion and metastasis of prostate cancer. ANXA2 can therefore be used as a prognostic biomarker for aggressive prostate cancer (Tan et al., 2021). ANXA9 expression levels have been correlated with the depth of invasion and lymphatic metastasis of colorectal cancer (Yu et al., 2018). Patients with colorectal cancer and positive ANXA9 expression were found to have a poorer prognosis, which indicates that ANXA9 could be an independent risk factor for survival. In extrahepatic cholangiocarcinoma, ANXA10 can induce tumour proliferation, EMT facilitation and tumour metastasis (Sun et al., 2019). A large number of preclinical and clinical studies have demonstrated that most ANXAs exhibit obviously invasive and metastatic properties. This may provide an explanation for cancer survival *via* the broken host immune defence and distant cancer growth. Accordingly, inhibiting ANXA-associated targets may offer new approaches to mitigate cancer metastasis and improve patient survival.

### 3.4 The role of ANXA protein family in cell apoptosis

**FIGURE 4:**
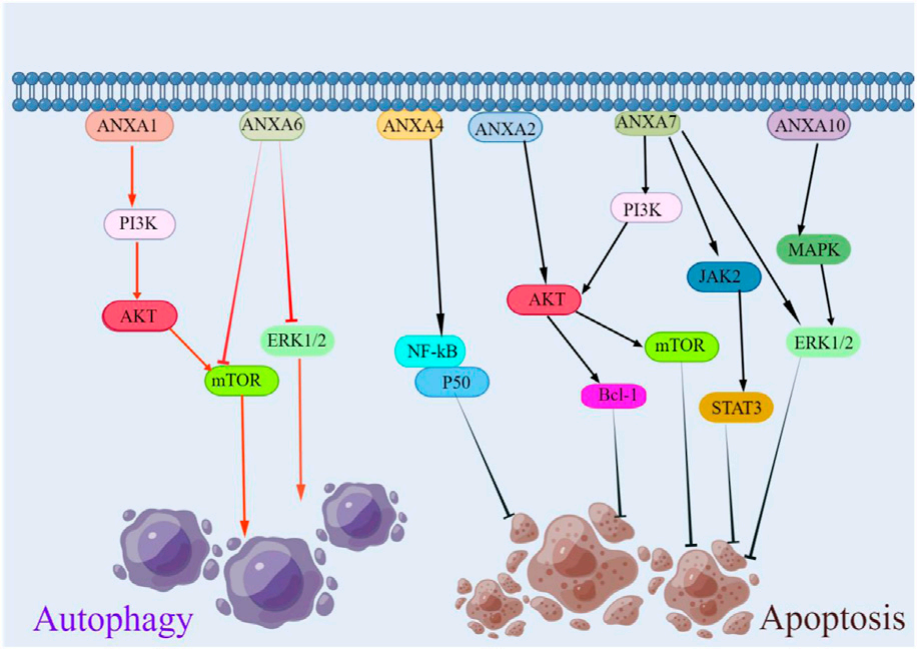
Regulation of ANXA protein family in apoptosis and autophagy. ANXA1 promoted cellular autophagy and ANXA6 inhibited cellular autophagy. In terms of apoptosis, except for ANXA10, which had a dual role, the rest of the ANXA protein family inhibited tumor cell apoptosis.

Apoptosis is an ordered and coordinated cellular process that occurs under physiological and pathological conditions (Wong, 2011). Evidence suggests that the ANXA protein family has a dual role in tumour cell apoptosis, namely, anti-apoptotic and pro-apoptotic activity (Figure 4) (Huang et al., 2008; Liu et al., 2012; Wei and Zhu, 2021). ANXA2 silencing significantly decreases the mRNA and protein expression of Bcl-1 and promotes apoptosis in osteosarcoma through the AKT signalling pathway and, in lung cancer, by the activity of p53 (Huang et al., 2008; Pan et al., 2018). The upregulation of ANXA4 enhances its interaction with NF-κB p50, activates the NF-κB signalling pathway, promotes cell cycle progression and inhibits apoptosis (Liu et al., 2020). ANXA7 knockdown reduces mRNA, the protein levels of LEPR and the intracellular signalling pathways of the ERK1/2, JAk2/STAT3 and PI3K-related proteins, thereby promoting apoptosis in hepatocellular carcinoma (Huang et al., 2021). While ANXA10 plays a diverse role in different tumours, in hepatocellular carcinoma, the overexpression of ANXA10 significantly promotes apoptosis. Conversely, in papillary thyroid cancer, the knockdown of ANXA10 promotes apoptosis by inhibiting the MAPK/ERK signalling pathway through the downregulation of TSG101 (Liu et al., 2012; Wei and Zhu, 2021). Although studies have shown the functional diversity of ANXA proteins in apoptosis, a deeper understanding of ANXA would make an important contribution to the field of cancer treatment by enhancing apoptosis.

### 3.5 The role of ANXA protein family in autophagy

Autophagy is a catabolic process involving the lysosomal renewal of proteins and organelles to maintain cellular homeostasis and reduce metabolic stress (Lozy and Karantza, 2012). Autophagy is one of the most important programmed cell death mechanisms, and the ANXA protein family has a considerable impact on autophagy (Figure 4). ANXA regulates the formation of vesicular lipid membranes and promotes cellular cytokinesis (Xi et al., 2020). Respective members of the ANXA protein family have different effects on autophagy in various cancers. ANXA1 inhibits cellular autophagy and promotes tumour invasion and metastasis through PI3K/AKT signalling activation in nasopharyngeal carcinoma (Zhu et al., 2018). In contrast, autophagy induced by ANXA6 in cervical cancer may be related to the inhibition of the PI3K-AKT and ERK signalling pathways of mTOR activation (Sun et al., 2020). Elucidating the molecular mechanism of ANXA in autophagy may assist in identifying new therapeutic targets and developing novel treatment strategies.

### 3.6 Summary

There is a growing consensus that ANXA proteins play a role in cancer and that the ANXA protein family is involved in tumour cell proliferation, cell cycle regulation, invasive metastasis, apoptosis and autophagy in numerous cancers (Table 1). The consensus thus far is that ANXA proteins in tumour cells may be mediators of tumorigenesis, proliferation and metastasis, that is, ‘angels of life’ for some tumours and ‘demons of death’ for others. This summary therefore provides an exciting opportunity for the treatment of various cancers.

**TABLE 1:** The role of ANXA family proteins in cancer progression.

Member	Cancer type	Type cell	Expression patterns	Effect	Reference
ANXA1	Thyroid carcinoma	TPC-1	mRNA and protein	High ANXA1 expression promotes the proliferation of thyroid cancer cells	Zhao et al., (2021)
	Nasopharyngeal carcinoma	5–8F 6–10B	Protein	ANXA1 promotes migration, invasion, and metastasis of nasopharyngeal carcinoma cells, and autophagy activation inhibits metastasis of nasopharyngeal carcinoma with high ANXA1 expression	Xiao et al., (2017), Liu et al., (2014) ^,^ Zhu et al., (2018)
	Hypopharyngeal carcinoma	FaDu	mRNA and protein	Silencing ANXA1 inhibited apoptosis and increased the proportion of S-phase cells	Li et al., (2021b)
	Leukemic	K562 U937	mRNA and protein	ANXA1 induces apoptosis and G_0_/G_1_ phase cycle arrest in K562 and U937 cells	Sabran et al., (2019)
	Breast cancer	168FARN	Protein	The expression of vimentin and myosin light chain kinase decreased after inhibiting ANXA1	Tsai et al., (2013)
ANXA2	Colorectal Cancer	ATCC	mRNA and protein	Significant reduction in growth and motility of colorectal cancer when ANXA2 expression was inhibited	He et al., (2019)
				Inhibition of ANXA2	
	Gastric carcinoma	HGC-27; MKN45	Protein	significantly reduces cell proliferation, migration and matrix metalloproteinase secretion	Han et al., (2017); Mao et al., (2021)
	Glioblastoma	Primary GBM cells	Protein	ANXA2 knockdown stalled the cell cycle in S-G2/M phase	Maule et al., (2016)
	Esophageal cancer	KYSE	Protein	Overexpression of ANXA2 activates the MYC-HIF1A-VEGF cascade through	Ma et al. (2018)
	Prostate cancer	Tissue	Protein	High expression of ANXA2 promotes invasion and metastasis	Tan et al. (2021)
	Hepatocellular carcinoma	Huh-7 QGY-7703; HepG2; SMMC-7721	mRNA and protein	TAGLN2-Annexin A2 interactions induce invasion and metastasis, shRNA-mediated ANXA2 silences inhibit proliferation, invasion, migration, and tumorigenic potential of hepatoma	He et al., (2019), Zhang et al., (2013), Shi et al., (2020)
	Lung cancer	BE1	Protein	Knockdown of ANXA2 promotes apoptosis and inhibits cell proliferation in lung cancer cells	Huang et al., (2008)
	Osteosarcoma	U-2 OS	mRNA and protein	ANXA2 promotes OS cell proliferation, migration, and invasion, and inhibits apoptosis	Pan et al., (2018)
ANXA3	Hepatocellular carcinoma	HepG2	Protein	ANXA3 promotes proliferation and angiogenesis of hepatocellular carcinoma	Guo et al., (2021a)
	Breast carcinoma	MDA-MB-231	Protein	After ANXA3 silencing, the proportion of G0 / G1 phase cells increased and cell proliferation rate decreased	Li et al., (2018b)
ANXA4	ovarian clear cell carcinoma	ES-2 RMG-1	Protein	ANXA4 promotes cell proliferation and inhibits cell apoptosis	Liu et al., (2020)
	Hepatocellular carcinoma	SMMC-7721	mRNA and protein	Down-regulation of ANXA4 expression inhibits hepatocellular carcinoma cell proliferation and tumorigenesis	Liu et al., (2017)
	Lung cancer	A549	Protein	ANXA4 is mediated by HPV16E7 and HPV16E6/E7, promotes cell proliferation, and regulates cell cycle and mitosis	Ciotti et al., (2009)
ANXA5	Cervical carcinoma	HeLa	Protein	Increased ANXA5 expression inhibits the proliferation and metastasis of cervical cancer cells	Li et al., (2018a)
ANXA5	Hepatocarcinoma	Tissue	Protein	overexpression promotes clinical progression and lymphatic metastasis in hepatocellular carcinoma	Sun et al., (2018)
	Colorectal cancer	ATCC	mRNA and protein	In colorectal cancer, ANXA5 is inhibited, resulting in G_1_ phase arrest	Xue et al., (2009)
ANXA6	Gastric cancer	MKN28	mRNA and protein	ANXA6 plays an oncogenic role in gastric cancer cells	Wang et al., (2013)
	Cervical cancer	HeLa	Protein	In cervical cancer, ANXA6 induces autophagy	Sun et al., (2020)
ANXA7	Hepatocellular carcinoma	Hca-F Hca-P	mRNA and protein	ANXA7 promotes the proliferation, migration and invasion of lymphatic metastatic cells from hepatocellular carcinoma and inhibits their apoptosis.	Huang et al. (2021)
ANXA8	Lung cancer	A549	Protein	Overexpressed ANXA8 promotes the proliferation of lung cancer cells	Zhou et al. (2021a)
ANXA9	Gastric cancer	HGC-27	mRNA and protein	Overexpression of ANXA9 increased the number of gastric cancer cells migrating	Zhou et al., (2021b)
	Colorectal cancer	Caco-2; HCT116; SW620	mRNA and protein	The expression level is correlated with the invasion depth and lymphatic metastasis of colorectal cancer	Yu et al., (2018)
ANXA10	Thyroid carcinoma	BCPAP; TPC-1; KTC-1	mRNA and protein	ANXA10 inhibits PTC cell proliferation and promotes PTC cell apoptosis by binding with TSG101	Wei and Zhu, (2021)
	Hepatocellular carcinoma	HepG2	mRNA and protein	Overexpression of ANXA10 gene inhibits proliferation and promotes apoptosis of HepG2 cells in vitro	Liu et al., (2012)
	Oral cancer	HSC-2; HSC-3; KON	mRNA and protein	ANXA10 promotes G_1_ phase cell cycle progression in oral cancer	Shimizu et al., (2012)
	Extrahepatic cholangiocarcinoma	QBC-939; FRH-0201	mRNA and protein	Induction of proliferation, EMT facilitation and tumor metastasis	Sun et al., (2019)
ANXA11	Hepatocellular carcinoma	Huh7; HCCLM3	mRNA and protein	ANXA11 promotes metastasis and the EMT pathway in hepatocellular carcinoma	Liu et al. (2019)

## 4 The role of ANXA protein family as a biomarker in cancer diagnosis

Previous studies have confirmed that the ANXA protein family is significantly associated with tumour development and can be used as a biomarker for cancer diagnoses. Through an *in vitro* study of 268 lung cancer patients, Rong et al. (2014) found that the expression of ANXA1 in the cancer tissues and serum of lung cancer patients was significantly higher than normal. Elevated serum ANXA1 was noted to closely correlate with the clinical manifestations in these patients. A large number of studies have also demonstrated that, in addition to lung cancer, ANXA1 expression is upregulated in a variety of cancers, including hepatocellular carcinoma (Zhuang et al., 2019), colorectal cancer (Liang and Li, 2021), pancreatic cancer (Novizio et al., 2021), melanoma (Delorme et al., 2021), and endometrial cancers (Aboulouard et al., 2021). By constructing a risk model, Liang and Li (2021) found that ANXA1 expression levels were associated with the level of immune infiltration in colorectal cancer. ANXA1 could therefore be used as a biomarker for colorectal cancer diagnoses and as an independent prognostic indicator for patients. Further, other ANXA protein family members have shown abnormal expression in many cancers. For example, the high expression of ANXA3 has been closely associated with upper tract urothelial carcinoma (Lu et al., 2014; Liu et al., 2021a), and patients with higher ANXA3 have been found to have a higher rate of postoperative recurrence (Liu et al., 2021a). Additionally, ANXA5 is significantly expressed in non-papillary bladder cancer (Wu et al., 2021), and lung cancer patients with high Cir–ANXA seven expression tend to have a poorer prognosis (Wang, 2021). Meanwhile, ANXA8 expression has been noted as significantly more enhanced in ovarian malignant tissues than benign tumours and normal ovarian tissues and is associated with a poor prognosis (Gou et al., 2019), and ANXA10 has recently been shown to be a prognostic biomarker for papillary thyroid cancer as well as a potential therapeutic target (Liu et al., 2021b). Similarly, ANXA13 expression is upregulated in colorectal cancer and promotes the invasion of cancer (Jiang et al., 2017). This suggests that ANXA has already been used as a diagnostic biomarker in a variety of cancers. More specifically, due to its unique structure, ANXA6 may act as either a tumour suppressor or a tumour promoter depending on the type and stage of cancer. ANXA6 expression has been reported to be upregulated in cancers such as pancreatic cancer (Leca et al., 2016), ovarian cancer (Noreen et al., 2020), female thyroid cancer (Lee et al., 2018), and esophageal adenocarcinoma (Zaidi et al., 2014), but downregulated in hepatocellular carcinoma (Meier et al., 2016), gastric cancer (Wang et al., 2013), breast cancer (Sakwe et al., 2011), cervical cancer (Lomnytska et al., 2010) and triple negative breast cancer (TNBC) (Korolkova et al., 2020). Such studies offer some important insights for the use of ANXA6 as a biomarker for the progression of these cancers (Table 2). In addition, ANXA1 expression is significantly increased in small cell lung cancer patients with bone metastases than without bone metastases (Chen et al., 2021). The elevation of ANXA1 has been detected in colorectal cancer patients with micro-metastases in the anterior lymph nodes than in matched individuals with normal lymph nodes (He et al., 2010). ANXA3 expression was also found to be higher in lung cancer patients with lymph node metastases than those without metastases. Accumulating evidence thus suggests that AXNA may be used as a potential biomarker for cancer metastasis (Liu et al., 2021a).

**TABLE 2:** The role of ANXA as a biomarker in cancer diagnosis.

Member	Cancer type	ANXA protein expression	Ref
ANXA1	Hepatocellular carcinoma	High expression	Zhuang et al. (2019)
	Colorectal cancer	High expression	Liang and Li (2021)
	Pancreatic cancer	High expression	Novizio et al. (2021)
	Melanoma	High expression	Delorme et al. (2021)
	Endometrial cancer	High expression	Aboulouard et al. (2021)
ANXA2	Lung cancer	High expression	Leca et al. (2016)
ANXA3	Upper tract urothelial carcinoma	High expression	Lu et al. (2014), Liu et al. (2021a)
ANXA5	Non-papillary bladder cancer	High expression	Wu et al. (2021)
ANXA6	Pancreatic cancer	High expression	Leca et al. (2016)
	Ovarian cancer	High expression	Noreen et al. (2020)
	Female thyroid cancer	High expression	Lee et al. (2018)
	Esophageal adenocarcinoma	High expression	Zaidi et al. (2014)
	Hepatocellular carcinoma	Low expression	Meier et al. (2016)
	Gastric cancer	Low expression	Wang et al. (2013)
	Breast cancer	Low expression	Sakwe et al. (2011)
	Cervical cancer	Low expression	Lomnytska et al. (2010)
	Triple-negative breast cancer	Low expression	Korolkova et al. (2020)
ANXA7	Lung cancer	High expression	Wang (2021)
ANXA8	Ovarian cancer	High expression	Gou et al. (2019)
ANXA10	Papillary thyroid cancer	High expression	Liu et al. (2021b)
ANXA13	Colorectal cancer	High expression	Jiang et al. (2017)

## 5 The role of ANXA protein family in cancer treatment

### 5.1 Role of ANXA protein family in therapy resistance

Therapy resistance is one of the fundamental reasons for cancer progression. It is primarily driven by tumour cells’ intrinsic mechanisms. Previous studies have shown that ANXA promotes therapy resistance to a large number of drugs, which suggests that ANXA plays an important role in treatment resistance. For example, ANXA4 was found to be overexpressed in paclitaxel-resistant lung cancer (Wei et al., 2012; Matsuzaki et al., 2014; Wei et al., 2015; Yao et al., 2016). In normal conditions, paclitaxel induces the translocation of ANXA4 to the cytoplasmic membrane and promotes apoptosis in cancer (Gaudio et al., 2013; Wei et al., 2015; Gaudio et al., 2016). However, ANXA4 can bind to the N-terminal part of the Fhit protein, a tumour suppressor gene, to form an ANXA4–Fhit complex, which specifically prevents cytoplasmic translocation and generates drug resistance (Huebner and Croce, 2001; Gaudio et al., 2013; Wei et al., 2015). Not only is the Fhit protein closely associated with ANXA4 and drug resistance, but also ATP7A. Toosendanin targets ANXA4/ATP7A to reduce the binding of ANXA4 to ATP7A and to mediate the sensitisation of non-small cell lung cancer to cisplatin (Zheng et al., 2018). Additionally, a vitro drug sensitivity assay demonstrated that the overexpression of ANXA3 significantly enhanced the resistance of hepatocellular carcinoma cells to cisplatin (Tong et al., 2015; Liu et al., 2021a), 5-fluorouracil (Pan et al., 2015; Tong et al., 2015; Liu et al., 2021a) and adriamycin (Liu et al., 2021a). A previous study found that the secretion of ANXA6-containing exosomes promoted paclitaxel resistance in breast cancer cells as well as cancer progression in a YAP1-dependent manner (Guo et al., 2021b). At the same time, reduced ANXA6 expression has been shown to sensitise TNBC cells to tyrosinase inhibitors (Korolkova et al., 2020), and Hsa-miR-105-1 expression can be downregulated in cisplatin-resistant ovarian cancer cells by targeting ANXA9 (Kou et al., 2021). The aforementioned information reveals a possible mechanism by which tumour cells become resistant to some anticancer drugs by affecting ANXA proteins.

Besides the traditional drugs mentioned above, some novel targeted drugs have been found to produce resistance by affecting ANXA proteins. Xiong et al. noted that upregulation of the ribonucleotide reductase M2 subunit (RRM2) causes resistance to sunitinib in renal cancer cells because it stabilises ANXA1 and activates the AKT pathway independently of its nucleotide reductase activity (Xiong et al., 2021). Spijkers-Hagelstein et al. found that ANXA2 was associated with glucocorticoid resistance in children with acute lymphoblastic leukaemia (ALL). The downregulation of ANXA2 expression significantly increased the sensitivity of ALL cells to glucocorticoids and prednisolone, and when ANXA2 was overexpressed in ALL cells, Src kinase activity was enhanced, which resulted in glucocorticoid resistance. Meanwhile, the downregulation of ANXA2 blocked this process, leading to increased sensitivity to prednisolone and thus improved therapeutic efficacy. Recently, ANXA2 has also been reported as a drug resistance-associated protein in ovarian cancer (Cruz et al., 2017). Furthermore, the knockdown of ANXA3 has been found to inhibit the resistance of hepatocellular carcinoma cells to sorafenib (Tong et al., 2018). The mechanism involved the overexpression of ANXA3 in sorafenib-resistant hepatocellular carcinoma cells, which inhibited PKCd/p38-associated apoptosis to promote cell survival. ANXA3 may therefore be important in the development of multidrug resistance in hepatocellular carcinoma patients. Although the mechanisms remain unclear, the anti-resistance function of ANXA sheds light on the utilisation of ANXA against drug resistance and potential improvements in treatment efficacy.

### 5.2 Role of ANXA protein family in modulation of therapy efficiency

As previously discussed, the ANXA protein family plays critical role in the development of cancer, so ANXA proteins have become viable new targets for cancer treatment. Natural compounds are one of the directions that may be used to effect the use of ANXA2 in treatment. For instance, the ginsenosides Rg5 and Rk1 can specifically bind to ANXA2. This interaction can inhibit NF-κB activity, downregulate apoptosis inhibitory proteins, activate caspases and promote apoptosis (Wang et al., 2018). Picrasidine, a plant alkaloid purified from the traditional Chinese medicine bitter ginseng, has been shown to achieve anti-tumour activity by targeting ANXA2 (Wang et al., 2017), and cicer arietinum lectin inhibits the EGFR -mediated signalling pathway by blocking the binding of ANXA2 and galactose lectin-3, with apoptosis as a consequence, and inhibiting cancer proliferation and migration (Shetty et al., 2016). A researcher designed a cationic lipid-directed nanoparticle, which is essentially an ANXA2 shRNA carrier, and found that it can suppress tumour growth by silencing ANXA2 (Andey et al., 2014). It has also been proposed that CBP12, a colorectal cancer binding peptide, can target ANXA2 with specific affinity and thus has the potential to become a novel targeted drug (Li et al., 2021c). In the meantime, Du et al. found that ANXA3 and HIF-1α act together to promote colon carcinogenesis, while HIF-1α siRNA can inhibit the expression of HIF-1α and ANXA3 in transplantation tumour tissues (Du et al., 2020), Liu et al. demonstrated that miR-1253 upregulates the expression of the pro-apoptosis-related proteins Bax and caspase-3 and downregulates the expression of the anti-apoptosis-related protein Bcl2 *via* the degradation of ANXA3 as its target, thereby promoting the apoptosis of cancer cells (Liu et al., 2021c).

To date, a negligible number of studies addressing this area of research have been published. Future research involving drugs that target ANXA will undoubtedly inspire the development of new strategies that enhance therapy efficiency.

### 5.3 The inhibitors of the ANXA protein family

Only a few studies are available on ANXA protein family inhibitors. The downregulation of ANXA expression mainly uses gene knockout and gene silencing. However, the identification of ANXA inhibitors would be beneficial as this would enable the targeting of ANXA proteins for cancer treatment. As described previously with respect to their structure, the functioning of ANXA proteins depends strictly on Ca^2+^, and some ion channel blockers exert an inhibitory effect on ANXA proteins (Kourie and Wood, 2000). In earlier studies, Pollard et al. found that concentrations of Ca^2+^ greater than 10 nM were able to block the synexin channel of ANXA7. While the non-activating Cd^2+^ channel inhibitor nifedipine required concentrations greater than 300 mM (Pollard and Rojas, 1988). Burns et al. pointed that the synexin channel of ANXA5 was sensitive to 0.2 nM La^2+115,116^. These ions and compounds inhibit ANXA function by blocking ion channels. Similarly, it was determined that benzothiazolecipine K201 could bind to ANXA5 and inhibit its Ca^2+^ channel activity because K201 was able to inhibit the hinge movement of the ANXA5 module in a metastable manner, thereby blocking Ca^2+^ movement in ANXA5 (Kaneko et al., 1997; Gerke and Moss, 2002). In a recent study, Aareskjold et al. synthesised RatRib120, a nuclease, with the ability to stably integrate with host DNA and enabled it to downregulate ANXA2 expression by targeting the ANXA2 mRNA (Aareskjold et al., 2019). In addition, ANXA8, which is highly expressed in patients with acute promyelocytic leukaemia, was found to be inhibited by all-trans-retinoic acid, an inhibitor that can help patients with symptom relief (Chang et al., 1992).

ANXA proteins characteristically bind to their receptors and relieve specific biological effects, so the inhibitors of ANXA receptors are key regulators of ANXA protein functions. n-t-Boc-Met-Leu-Phe is a classic ANXA1 receptor blocker (Liu et al., 2015; Vago et al., 2016; Bai et al., 2020) that competitively binds to the FRP2 receptor (Bai et al., 2020). In addition, due to the function of the interaction between ANXA and S100A10 in cancer, Reddy et al. screened 29 potential inhibitor compounds and determined that 2-[5-(4,6-dimethyl-pyrimidin-2-ylsulfanylmethyl)-4-furan-2-ylmethy l-4H-[1,2,4]triazol-3-ylsulfanyl]-N-substituted (R)-acetamide analogues and 3,4,5-trisubstituted-1,2,4-triazole analogues were the two inhibitors that attached to the N-terminal of ANXA2 (Reddy et al., 2014). This attachment not only disrupted the interaction between ANXA and S100A10, but also inhibited ANXA2 activity.

A recent study found that phenothiazine derivatives, especially trifluoperazine (TFP), are able to inhibit the repair function of ANXA proteins at the cell membrane. TFP delays the accumulation of ANXA2-GFP at the damaged plasma membrane, inhibits ANXA-mediated membrane curvature, affects the membrane-forming ability of ANXA and, to some extent, impairs ANXA proteins from binding to membrane patches (Table 3) (Heitmann et al., 2021). Not only inhibitors, but also their functions need deeper investigation. It remains an open question whether inhibitors are a potential target for cancer treatment and further, how to transfer this knowledge to clinical application.

**TABLE 3:** Inhibitors of ANXA family protein.

Member	Inhibitors	Inhibitor type	Effect	Ref
ANXA1	N-t-Boc-Met-Leu-Phe(BOC-1)	Compound	Binds competitively to FRP2 receptors	Liu et al. (2015), Vago et al. (2016), Bai et al. (2020)
ANXA2	RatRib120	Nucleases	Target ANXA2 mRNA	Aareskjold et al. (2019)
	2-[5-(4,6-dimethyl-pyrimidin-2-ylsulfanylmethyl)-4-furan-2-ylmethyl-4H-[1,2,4]triazol-3-ylsulfanyl]-N-substituted (R)-acetamide analogues and 3,4,5-trisubstituted-1,2,4-triazole analogues	Compound	Acts on the N-terminal of ANXA2 to inhibit the inhibitor compound of ANXA2 bound to S100A10 and inhibits ANXA2 itself	Reddy et al. (2014)
ANXA5	Benzothiazolecipine K201	Compound	Binds to ANXA5 and inhibits its Ca^2+^ channel activity	Kaneko et al. (1997), Gerke and Moss (2002)
	La^2+^	Cation	block the synexin channel of ANXA5	Rojas et al. (1990)
ANXA7	La^2+^	Cation	block the synexin channel of ANXA7	Burns et al. (1989)
	Cd^2+^(>10 nM)	Cation	block the synexin channel of ANXA7	Pollard and Rojas (1988)
	Nifedipine (>300 nM)	Compound	block the synexin channel of ANXA7	Pollard and Rojas (1988)
ANXA8	all-trans-retonoic acid	Compound	Down-regulation of highly expressed ANXA8 in acute promyelocytic leukemia patients	Chang et al. (1992)
Pan-inhibitors of ANXA	Derivatives of phenothiazine, especially trifluoperazine (TFP)	Compound	Inhibits the repair function of cell membrane or plasma membrane by impairing the function of ANXA	Heitmann et al. (2021)

## 6 Conclusion and future directions

Most of the numerous members of the ANXA protein family have been found to be associated with cancer, which is characterised by abnormal changes in the normal cellular pathway through the up- or downregulation of expression. Due to the individual characteristics of tumour cells and the various post-translational patterns of the ANXA protein family, members of the ANXA protein family trigger different tumorigenesis and metastasis, thus leading to variable outcomes. A more in-depth study of the ANXA protein family in the tumour microenvironment, with careful consideration of the timing and approach to target selection, may suggest more biologically meaningful clinical treatment strategies. In the early stages of cancer, high expression of ANXA proteins may promote metastasis. It is therefore important to consider the timing of the use of ANXA proteins as clinical prognostic biomarkers. The expression of ANXA proteins is significantly altered during cancer progression, so they can be used as diagnostic and prognostic biomarkers for certain cancers. Furthermore, targeting ANXA proteins has emerged as a promising new strategy for cancer therapy. ANXA proteins are also closely associated with multidrug resistance in many cancers, including cisplatin, paclitaxel and sorafenib resistance. If this could be a research direction and the sensitivity of cancer cells to drugs could be improved, it would undoubtedly be of significant help in cancer treatment. Currently, most of the drug studies involving ANXA proteins are still in the laboratory stage with only a few clinical applications. How to apply these findings to clinical treatment is thus one of the future challenges in cancer research. Basic research on the ANXA protein family is currently relatively shallow, and many members of this protein family have not been studied in an integrated manner with the aim of enhancing tumour prevention and treatment. Future research should focus on the role of the ANXA protein family in the tumour microenvironment and consider tumour-related macrophages, tumour-related fibroblasts and lymphatic metastasis to achieve the combination of cancer prevention and treatment. Moreover, such research should focus on drug screening, as well as drug resistance and its role in immune suppression mechanisms, and aim to achieve precise treatments.

## Abbreviations

ALL, Acute lymphoblastic leukemia; ANXA, Annexin A; ANXs, Annexins; ATP, Adenosine triphosphate; Bcl-2, B-cell lymphoma-2; EGFR, Epidermal growth factor receptor; EMT, Epithelial-mesenchymal transition; ERK, Extracellular signal-regulated kinase; FHIT, Fragile histidine triad; GBM, Glioblastoma multiforme; HIF, Hypoxia Inducible Factor; HepG2, Human hepatocellular carcinomas; HPV, Human papillomavirus; IL-6, Interleukin-6; JAK2, Janus-family tyrosine kinase 2; MAPK, Mitogen-activated protein kinase; MEK, MAPK/ERK kinase; MMPs, Matrix metalloproteinases; MYC, Myelocytomatosis oncogene cellular homolog; PI3K, Phosphoinositide 3 kinase; AKT, AKT8 virus oncogene cellular homolog; RRM2, Ribonucleotide reductase M2 subunit; STAT3, Signal transducer and activator of transcription 3; TAGLN, Transgelin; NF-κB, Nuclear factor κ B; TFP, Trifluoperazine; NBC, Triple negative breast cancer; TSG, Tumor necrosis factor-stimulated gene; VEGF, Vascular endothelial growth factor.
